# The all-or-none repolarization in cardiac ventricular myocytes: an *in silico* characterization of a relevant biomarker of ventricular action potential

**DOI:** 10.3389/fphys.2026.1827577

**Published:** 2026-06-30

**Authors:** Massimiliano Zaniboni

**Affiliations:** Department of Chemistry, Life Sciences and Environmental Sustainability, University of Parma, Parma, Italy

**Keywords:** all-or-none repolarization, biomarker of cardiac repolarization, cardiac action potential form, cardiac action potential repolarization, discontinuous conduction of cardiac action potential, gradients of repolarization

## Abstract

All-or-none repolarization (AONR) consists in the fact that brief hyperpolarizing current injections delivered during the early phase of the cardiac action potential (AP) can reach membrane potential values where small changes in the current can either lead the post-injection membrane potential to recover the previous AP waveform or suddenly repolarize to the resting state. The phenomenon, long known from the early studies on squid axon and Purkinje fibers, has been studied over the years. Here, by means of an updated human ventricular AP model, the form of AONR is described in detail, showing how it is modified by the pacing rate and by the changes in the ion channels active during the early phase of AP. A novel representation of AONR is proposed that makes it easier to appreciate the features of this phenomenon and its relationship with the corresponding AP. It is shown, for example, how the threshold for AONR changes in different models of human ventricular AP. The phenomenon is revisited by which different propensity to generate AONR in endocardial and epicardial cells can lead to transmural repolarization gradients and arrhythmias. It is also shown that the form of AONR is mostly independent from the corresponding AP waveform, as two almost identical AP waveforms show completely different thresholds for AONR. The main message of this study is that the threshold for AONR is an easily accessible feature of the AP, rich in information concerning AP dynamics, which should be taken into consideration as a fundamental electrophysiological biomarker in the process of fine-tuning human ventricular AP models.

## Introduction

The fact that a brief polarizing current injection during the early phase of the cardiac action potential (AP) could, when released, return the AP to its unperturbed waveform or abruptly repolarize the membrane potential to its resting state, thus interrupting the AP, depending on the amount of injected current and the time of injection, has been observed since the early years of cardiac cellular electrophysiology. Silvio Weidmann was the first to show this effect on sheep Purkinje fibers (Weidmann, 1951) and Cranefield and Hoffman in papillary muscle preparation (Cranefield and Hoffman, 1958). The effect was shown in the same years also in non-cardiac preparations such as the squid axon by Huxley (Huxley, 1959) and was described in its biophysical mechanism by Noble and Hall (Noble, 1962a, 1962; Hall and Noble, 1963). A threshold for all-or-none repolarization (AONR) was described in the early phase of AP, which shifted from very polarized membrane potential values to more depolarized ones as the time of injected currents increased (Vassalle, 1966). Trenor and co-authors have recently reviewed AONR studies (Trenor et al., 2017). Since the early studies, an alternative method to characterize the threshold for AONR has been adopted, where instead of injecting current, the membrane potential during early AP repolarization was clamped for a few milliseconds (1–20 ms) at progressively polarized potential values and, after the release of the voltage clamp, the trajectory of the membrane potential was examined, determining the voltage clamp value that induced AONR (Vassalle, 1966; Beeler and Reuter, 1977). AONR has been found to have the features of a self-regenerative voltage displacement exactly like the initiation of AP, the first repolarizing and the second depolarizing, and early and recent studies have investigated its propagation in excitable media such as axon fibers or cardiac tissue (Noble and Hall, 1963; Hall and Noble, 1963; Himeno et al., 2023). Besides the intrinsic relevance for the dynamics of the cardiac AP, AONR has assumed over the years significance also for what concerns the initiation of arrhythmias. It has been found that AONR is more likely to happen in epicardial than endocardial myocytes, giving rise, when it occurs, to a large dispersion of repolarization across the ventricular wall, leading to electrocardiographic elevation of the ST segment (the so-called idiopathic J wave), which can in turn result in phase 2 reentry, and extra systolic beats potentially precipitating into tachycardia or fibrillation (Antzelevitch et al., 1999). For the same reason, the epicardium and the endocardium are differently affected by sodium current blockade (Krishnan and Antzelevitch, 1991), where, in fact, the epicardium, expressing more *I*_to_, is more prone to AONR whenever the sodium current is diminished or blocked. Along the same line, mutations in the *SCN5A* gene found in Brugada syndrome cause a reduction in the sodium current and, in turn, the AONR in the epicardium and not in the endocardium, leading to a gradient of repolarization that favors reentry (Antzelevitch, 2001). Thus, dispersion of repolarization due to different propensity to show AONR has been documented in many different pathological conditions (Di Diego and Antzelevitch, 1993, 2003; Dong et al., 2006, 2011). Despite the relevance of AONR in the physiology of cardiac excitation and its involvement in arrhythmia development, and despite all in-depth studies on its mechanism and propagation, there is a lack of a systematic description on the profile of AONR with respect to the AP in control and under modulation of the ion currents. The present study aimed to graphically represent the threshold for AONR, explain its features and its differences in different physiopathological conditions, and explain what is required to have AONR and how its shape affects AP conduction. Most of all the aim of this work was to show that the profile of the AONR threshold is a key determinant of AP dynamics, an easy-to-derive biomarker that should be taken into consideration in the process of tuning cardiac AP models to achieve a closer definition of their natural dynamics.

## Methods

### Modeling and simulation framework

The simulations presented in this study have been performed with the Tomek human ventricular AP model (Tomek et al., 2019), which is one of the most adopted and updated formulations to reproduce human ventricular electrical activity. The ten Tusscher and Panfilov model (ten Tusscher and Panfilov, 2006) and the Iyer, Mazhari, and Winslow (IMW) 2004 model (Iyer et al., 2004) have also been used. All models have been downloaded from the Cellml Repository (https://models.cellml.org/electrophysiology). The stiff ‘ode15s’ solver built into the R2025b version of Matlab (The MathWorks, Inc., Natick, MA, USA) was used to integrate the model’s equations. All simulations were run on a personal computer (PC) with Intel® Core^TM^ i7, 2.8-GHz CPU.

### Stimulation protocols

APs were elicited by simulating 0.5-ms-long current injections with an amplitude 50% above the current threshold. AP duration (APD) was measured as the time between the maximum first derivative of the membrane potential (*V*_m_) during the initial fast depolarization phase and the time during repolarization when *V*_m_ reached the value of −60 mV. The initial conditions at different basic cycle lengths (BCLs), or for any modulation of the maximum conductance of the ion channels, were measured after 1,000 beats conditioning pacing. When not differently specified, APs were measured at their stationary form at BCL = 2,000 ms. In addition, the modulations of the ion currents were made by conditioning pacing trains of 1,000 beats in order to reach steady-state APD configuration under the modified conditions. Thus, at *T* = 0 in each simulation, the ion current modulation was already at its steady-state condition.

### All-or-none-repolarization

**Figure 1:**
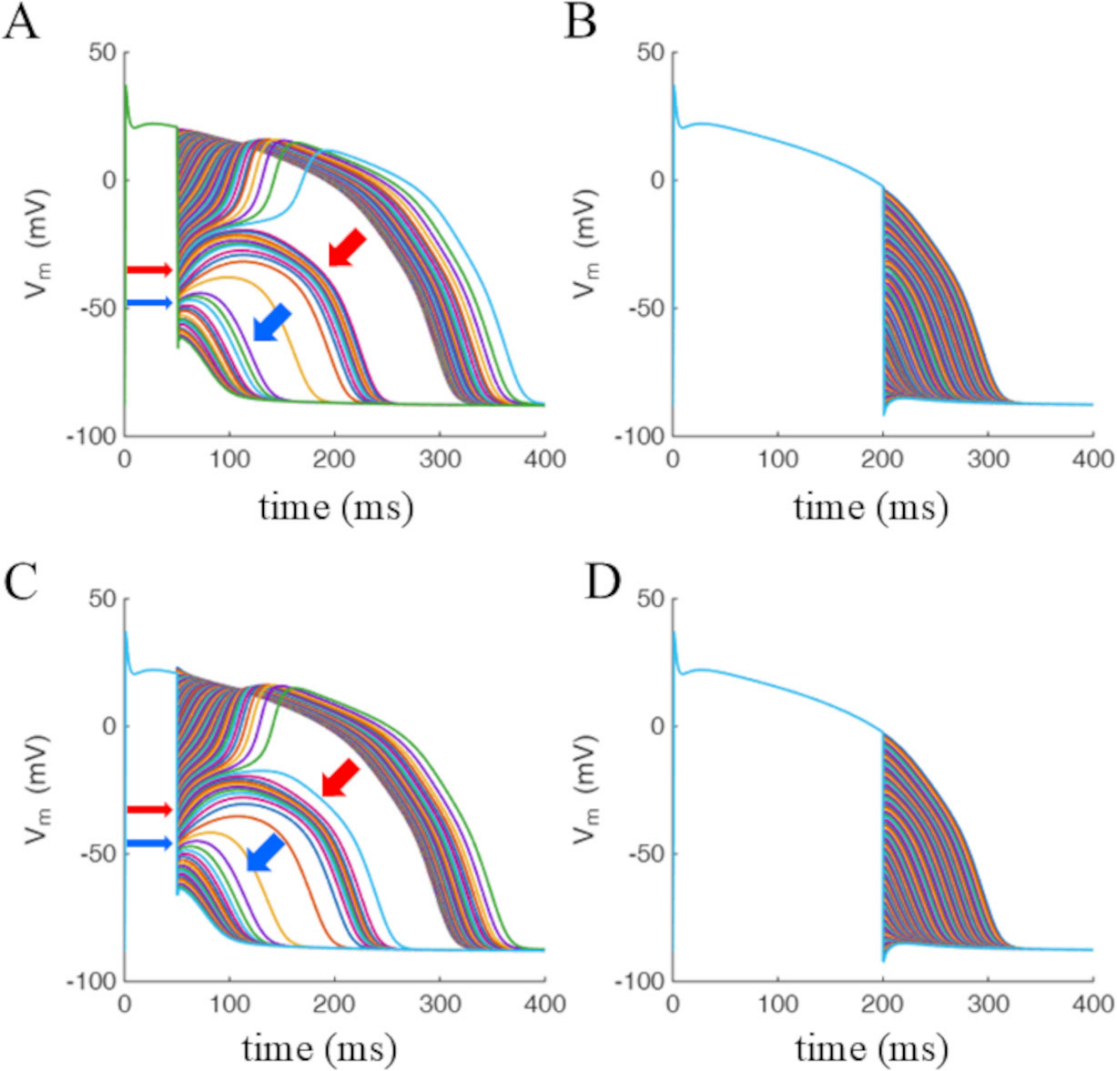
All-or-none-repolarization with current and voltage clamp. **(A)** during the AP repolarization, starting from time T = 5 ms and for time-increments of 5 ms, 1 ms current injections of increasing amplitude were delivered across the membrane. The example reported in the panel corresponds to time T = 50 ms. As the injected current increases linearly, the post-injection AP trajectories undergo continuous changes until two current values (small arrows) are reached where a small current increment corresponds to a qualitative change in the form of post-injected waveform (large arrows). **(B)** Later during AP repolarization, for example at T = 200 ms, as the injected current increases linearly the post-injection AP waveform changes continuously. **(C)** The same result is obtained when, at the same times T described above, the membrane is clamped for 1 ms at progressively polarizing increasing values, step 1 mV, from the value of V_m_ occupied by the AP at that time T. In this case, two values of V_m_ can be identified (small arrows) where, as the clamped Vm increases of 1 mV, the post-clamp AP trajectory changes qualitatively (large arrows). **(D)** When the voltage clamped steps are applied later during the AP repolarization, here at time T = 200 ms, no discontinuities appear in the post-clamp AP trajectories.

It is known that, when a brief polarizing current pulse is injected across the membrane during the early phase of a ventricular AP, as the amplitude of the current pulse increases linearly, for given values of current amplitude, the post-injection trajectory of AP undergoes sudden highly nonlinear changes (red and blue arrows in **Figure 1A**); that is, for certain current amplitudes, a small increase in current leads to dramatic qualitative changes in the AP trajectory. At later times during AP repolarization, this nonlinear behavior fades away and the AP trajectories change smoothly as the current injection increases (**Figure 1B**). The same behavior can be obtained as is simulated in **Figures 1C, D**, where, at a given time, *T* (50 ms in the figure), the membrane potential is voltage clamped (for 1 ms in this case) at progressively more polarized values and then the clamp is suddenly released. Similarly, as in the current injection experiment, the released voltage traces encounter discrete changes (red and blue arrows) for given values of clamped voltages when the voltage clamp is performed in the early phase of AP repolarization (**Figure 1C**); otherwise, they change smoothly to faster repolarization trajectories (**Figure 1D**).

### Graphical detection of the threshold for AONR

**Figure 2:**
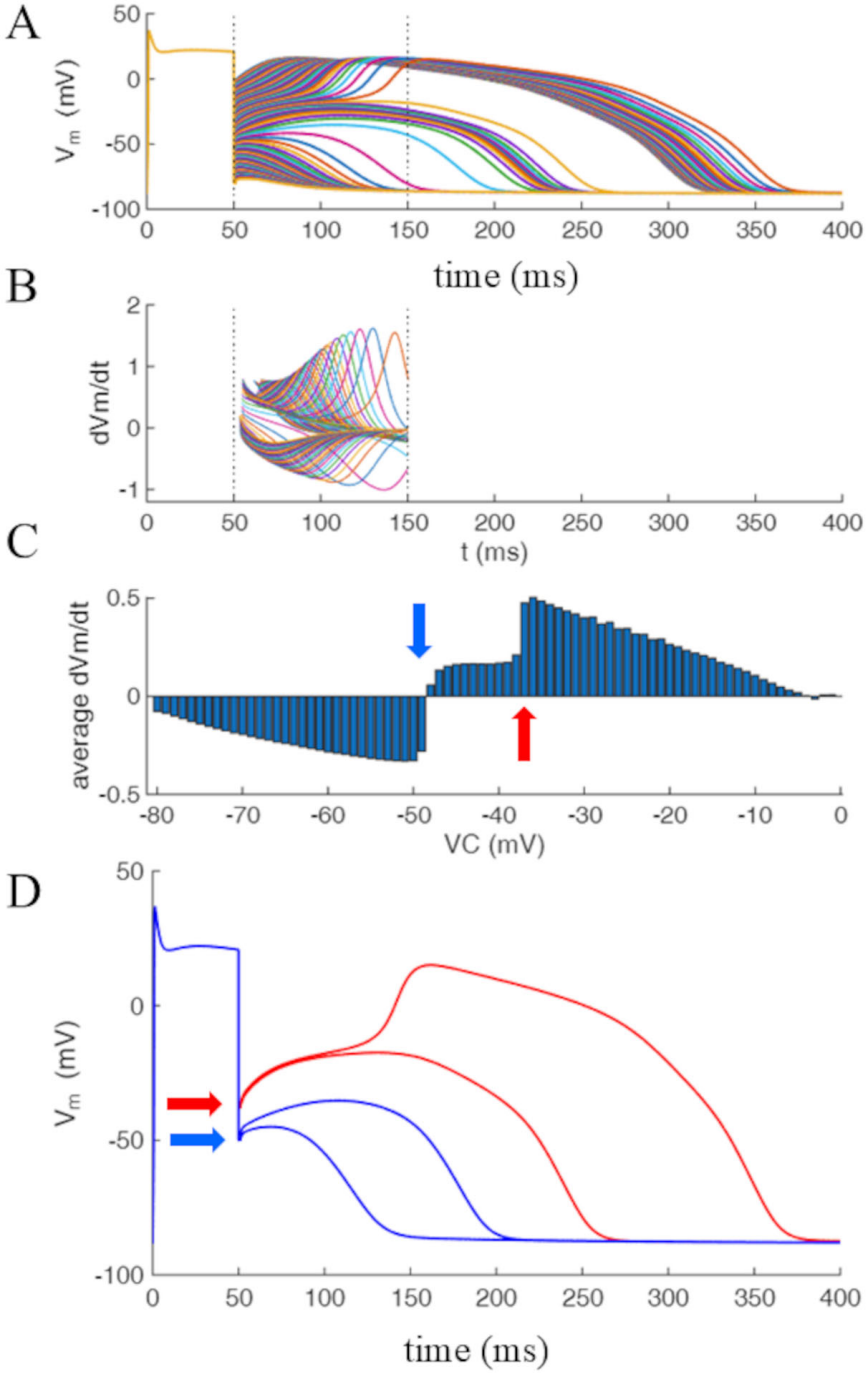
Graphical identification of repolarization discontinuities. **(A)** The volage clamp protocol described in **Figure 1C** is applied here at time T = 50 ms. **(B)** the first time-derivative of each post-clamp voltage trace is measured for a time window of 100 ms. Each derivative corresponds to a voltage-clamped value of V_m_. **(C)** The averaged value of each time-derivative is reported here as a function of the corresponding clamped voltage. The profile of this histogram highlights two discontinuities (blue and red arrows), which correspond to the voltage values where a 1 mV incremefnt leads to a qualitative change in the post-clamp AP profile [same arrows in panel **(D)**].

In the same simulation in **Figure 1C**, the averaged value of the time derivative of each voltage deflection was derived, according to the fundamental theorem of calculus, as the difference between the final and initial values of *V*_m_ deflections divided by the time interval (100 ms). The average value of the time derivative was reported as a function of the corresponding clamped voltage value in **Figure 2C**. Two discontinuities are evident in this type of representation, which correspond to the voltage values (see arrows) where discontinuous changes in the voltage trajectory take place (**Figure 2D**). This method makes less arbitrary the detection of the voltage clamp values where sudden changes appear in the AP trajectory, particularly at later times during AP repolarization. It must be noted that the time window for measuring the first derivative, here chosen as 100 ms, is arbitrary, although the results do not change qualitatively for slightly smaller or longer values. Moreover, due to the first derivative of the membrane potential (changed in sign) being proportional to the total ion current flowing across the membrane within the chosen time window, its measure retains the physical significance of a measure of membrane current.

### 3D representation for AONR thresholds

**Figure 3:**
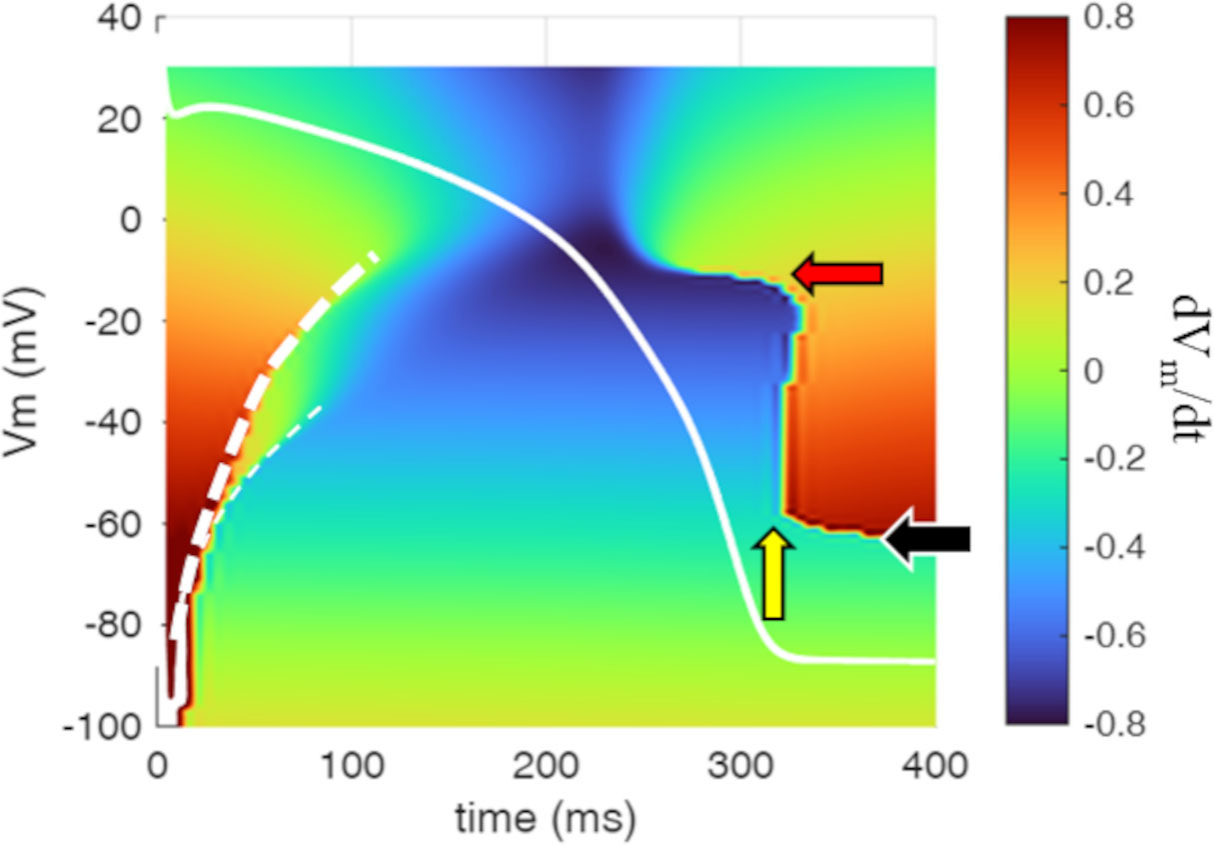
A three-dimensional surface describes thresholds for AONR. The average derivative profiles, like those measured in **Figure 2C**, are time-aligned every 5 ms from the onset of the AP up to time = 400 ms, originating a surface whose color, at each time and each voltage, is the average time-derivative of the post-clamp AP trajectory. The surface shows the threshold for AONR (thick dotted line). An additional secondary threshold is described by the thin dotted line. The surface also shows the threshold for calcium current (magenta arrow), the end of absolute refractory period (green arrow), and the threshold for fast Sodium current in diastole (black arrow).

When the protocol described in **Figure 2** is performed at increasing times during the AP (in the simulation in the figure from time 0 to 400 ms, with a 5-ms step) and the results similar to those in **Figure 3B** time-aligned along the AP time, a three-dimensional representation similar to that in **Figure 3** is obtained, where the AP time is reported on the *x*-axis, the membrane voltage on the *y*-axis, and the averaged value of the first derivatives on the *z*-axis in color (see color bar on the right). The AP waveform is superimposed in white color. In this representation, the voltage threshold of AONR can be seen as a color discontinuity along the thick broken white line (corresponding to the red arrows in **Figure 2**). The second discontinuity is marked as a thin broken white line in the same figure (blue arrow in **Figure 2**). I note in passing that, in the same figure, the threshold for *I*_CaL_ activation (red arrow), that for *I*_Na_ activation (black arrow), and the end of the absolute refractory period (yellow arrow) can also be identified. Henceforth, I will call threshold for AONR the one marked with the thick line, as the second one is not always present and has not been described in real experiments.

**Figure 4:**
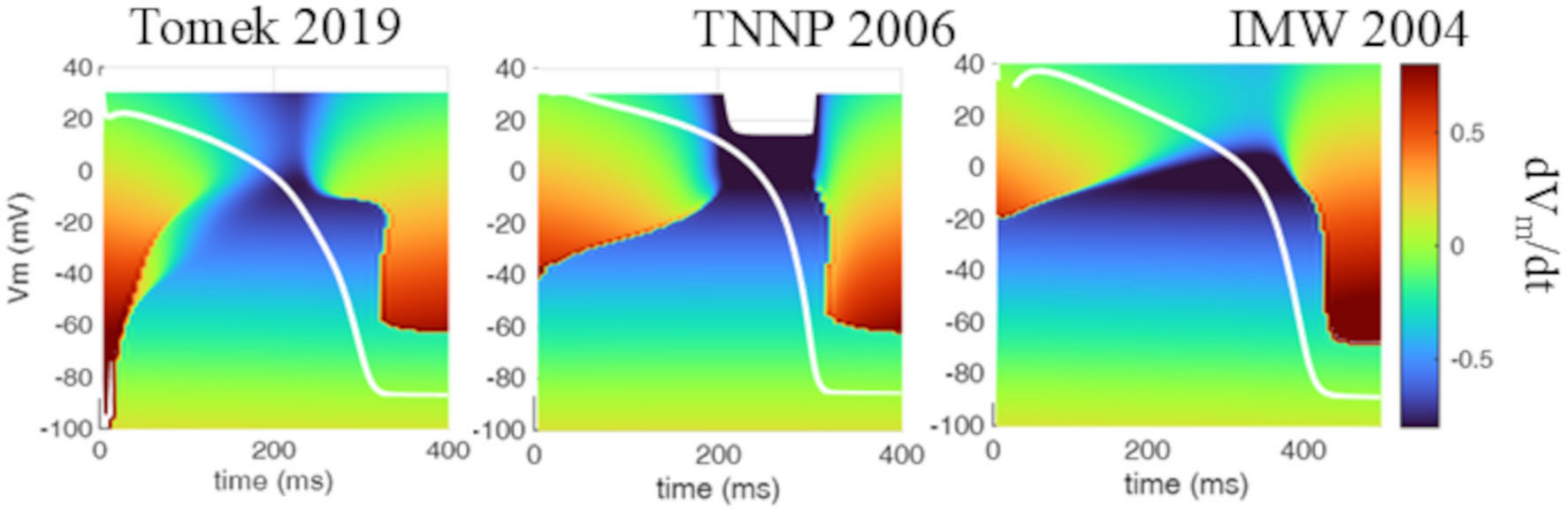
Different human ventricular models. The same surface described in **Figure 3** was measured for the Tomek 2019 model (left), the TNNP 2006 model (middle), and the IMW 2004 model, all at BCL = 2000 ms. To note, the dramatic differences in form and duration of the thresholds for AONR in the three human AP models.

The same representation of **Figure 3** was reproduced for two additional human ventricular AP model formulations, the ten Tusscher and Panfilov model (ten Tusscher and Panfilov, 2006) and the IMW 2004 model (Iyer et al., 2004), and reported in **Figure 4**. It should be noted that, even in models that share common dynamic features such as the APD rate dependence, the AONR profile is very different in the spanned voltage range and in the duration compared with APD. This might be due to the different composition in the ion currents of the three models, particularly in the relative contribution of *I*_Kr_ and *I*_Ks_. Note that the Tusscher–Noble–Noble–Panfilov (TNNP) and IMW models lack the second threshold marked with the thin line in **Figure 3**, as they lack the late sodium current *I*_NaL_ in their formulation, which, as will be shown later, is responsible for this threshold.

**Figure 5:**
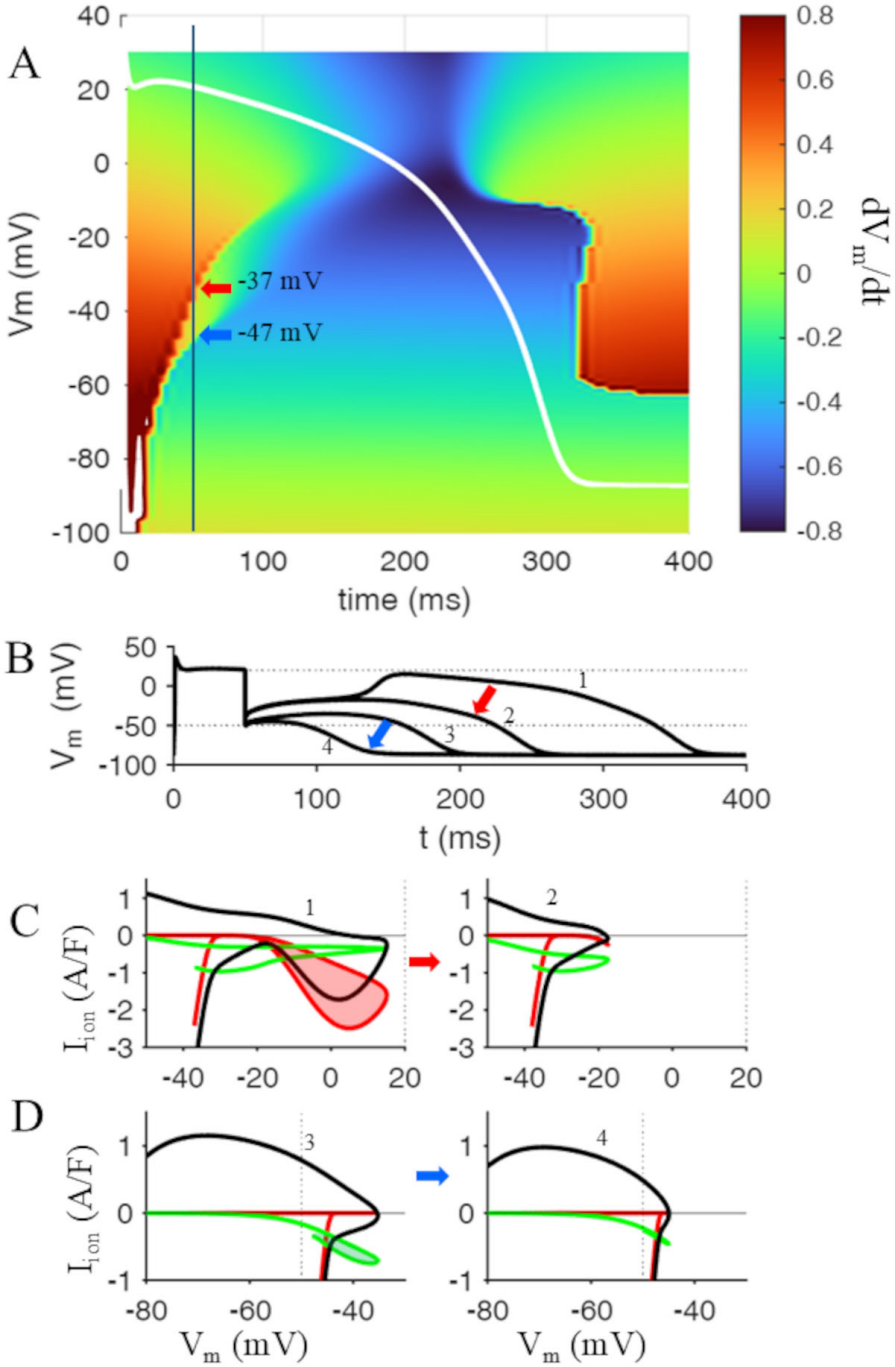
*I*_CaL_-mediated and *I*_NaL_-mediated AONR thresholds. The ion currents responsible for the two thresholds for AONR found in the Tomek 2019 model are studied at time T = 50 ms **(A)**. At the clamped value of V_m_ = -37 mV it is enough adding 1 mV in polarizing direction for the post-clamp V_m_ trajectory passing from curve 1 to curve 2 **(B)**. The same is true at V_m_ = -47 mV where 1 mV difference brings the V_m_ trajectory from curve 3 to curve 4. In panels **(C, D)** these transitions are seen in the phase plots where total ion current (black), *I*_CaL_ current (red), and INaL current (green) are recorded during the post-clamp time and reported versus V_m_. The transition from curve 1 to 2 **(C)** coincides with failure of *I*_CaL_ to reach its threshold and re-activate. The closed loop described by *I*_CaL_ activation is filled in red to emphasize the difference. Similarly, the transition from curve 3 to curve 4 (panel **D**) coincides with failure of *I*_NaL_ to reach is threshold and re-activate. Also in this case the loop described by INaL activation is filled in green.

Regarding the ionic mechanism underlying the threshold for AONR, an example with the Tomek model is reported in **Figure 5**. If we analyze the color map (**Figure 5A**) , for example at *T* = 50 ms of the AP, the two thresholds appear (red and blue arrows), which correspond to the discrete transitions in the voltage trajectories reported in **Figure 5B**. **Figures 5C, D** report, for each voltage displacement, the phase plot (membrane ion current *vs*. membrane potential) for the total ionic current (black), the *I*_NaL_ current (green), and the *I*_CaL_ current (red). **Figure 5C** clearly shows that the first threshold (red arrow) is due to failure to reactivate, when passing from *V*_m_ = −37 mV to *V*_m_ = −38 mV, of *I*_CaL_, which is sustaining the depolarization of trace 1. In the case of the second threshold (blue arrow), once again, a 1-mV difference (from −47 to −48) in the clamped potential leads to failure to reactivate of *I*_NaL_ (see **Figure 5D**), which is therefore the one responsible for the qualitative difference between trace 3 and trace 4. In both instances, among the currents responsible for AONR, the first, already active at both threshold values, is *I*_Kr_, followed by *I*_K1_ at more polarized potentials, both characterized by a negative-sloping *IV* curve in this voltage range and therefore leading to self-regenerative repolarization. The role of *I*_Ks_ in the Tomek 2019 model, as in the majority of models, is almost negligible compared with that *I*_Kr_ and *I*_K1_. Other ion currents have been analyzed, but are not considered here since their changes are continuous as opposed to the all-or-none behavior of *I*_CaL_ and *I*_NaL_.

## Results

### Role of ionic currents in modulating the threshold for AONR

**Figure 6:**
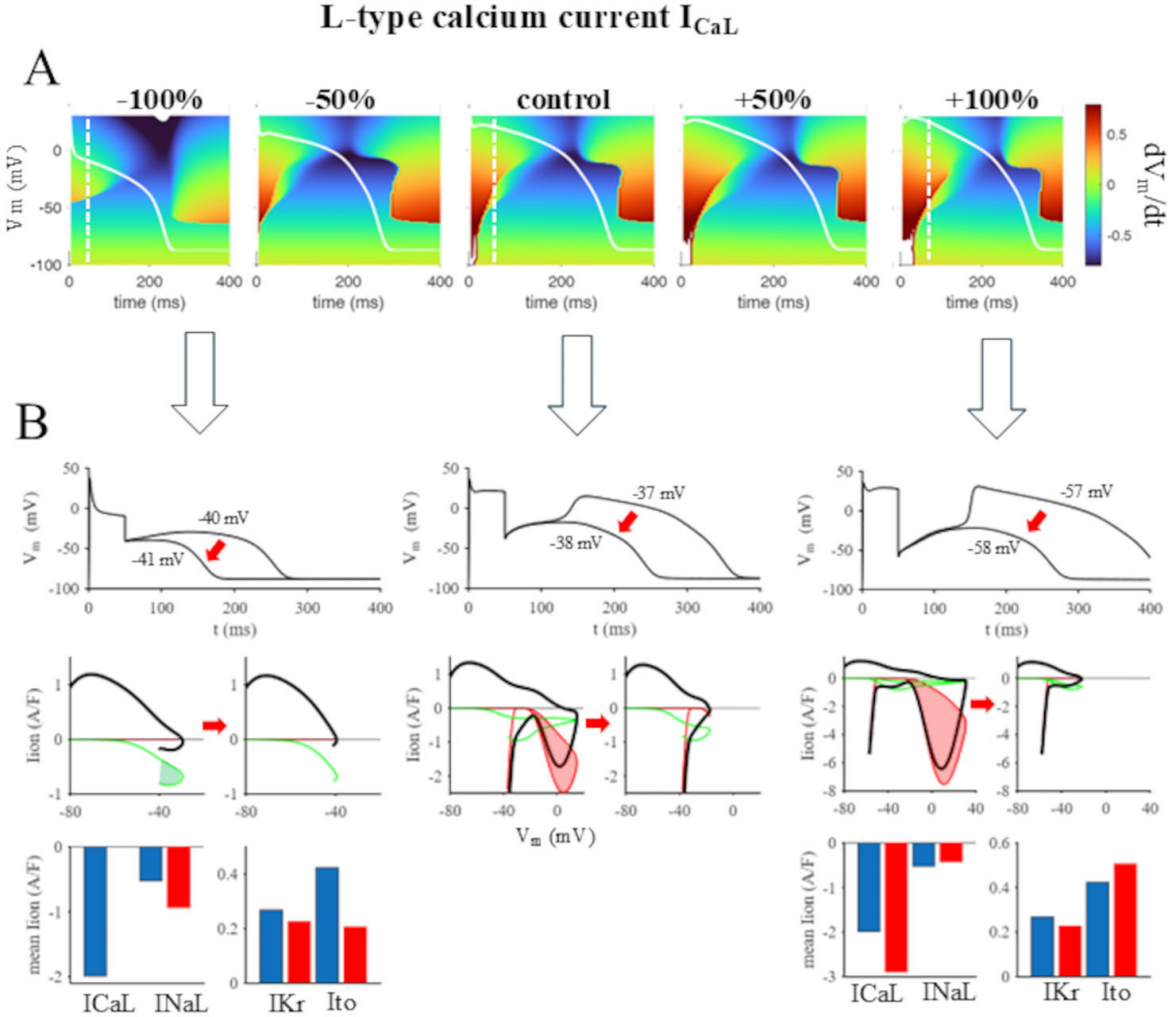
*I*_CaL_-modulation of AONR threshold. Panels in **(A)** represent how the complete abolition or the 50% decrease of *I*_CaL_, or its increase by 50 or 100% modify the form of AONR thresholds. Corresponding AP waveforms are reported superimposed to each surface. The analysis of thresholds is also performed in panel **(B)** for -100%, control, and 100% changes. In the case of complete abolition of *I*_CaL_ an AONR threshold still remains, due to *I*_NaL_, like explained in middle left panel **(B)**. In the lower panels, histograms show average value of depolarizing (left) and polarizing (right) currents in the 5 ms preceding the voltage-clamp (in blue the control values, in red the values after I_CaL_ modulation). The complete removal of I_CaL_ causes an increase in pre-clamp I_NaL_ and a decrease in pre-clamp *I*_Kr_ and *I*_to_. The AONR threshold at V_m_ = -40 mV is in this case all due to *I*_NaL_. In the control only the threshold at V_m_ = -37 mV due to I_CaL_ is considered. In the case of 100% increase of *I*_CaL_, in order to switch off *I*_CaL_ re-activation, the clamped Vm has to reach the very polarized value of -58 mV. Furthermore, the large depolarization due to *I*_CaL_ increase leads to a decrease of pre-clamp *I*_Kr_. Thus, *I*_K1_ is the major responsible for terminating the AP in this case.

In the following simulations, the maximum conductance of the different ion currents was modified and the APs were made to reach their steady state with a conditioning train of 1,000 beats. **Figure 6A** reports the color maps measured, always at BCL = 2,000 ms, after the complete block of *G*_CaL_ (first panel), after its reduction by 50% (second panel), the control map (third panel), the increment of *G*_CaL_ by 50% (fourth panel), and by an increment of 100% (fifth panel). The corresponding AP waveforms, modified rate-dependently, are superimposed in each panel. The measures reported in **Figure 6B** are performed at *T* = 50 ms of the AP and made only for the first, third, and fifth panels of **Figure 6A**. In the absence of *I*_CaL_, the only threshold for a depolarizing current is that of *I*_NaL_, which is in fact responsible for the discrete transition at *V*_m_ = −40 mV (left panel in **Figure 6B**). The bar plot at the bottom reports the average values of the main depolarizing (left) and repolarizing (right) currents in the 5 ms preceding the voltage clamp, with blue as the controls and red as the test. In this first example, of course *I*_CaL_ is zero, although it is notable that, in the absence of *I*_CaL_, the relatively polarized early plateau phase brings about a significant increase in *I*_NaL_ and a decrease of *I*_Kr_ and *I*_to_ (see bar plot). Note that, in the absence of *I*_CaL_, the only threshold in the depolarizing direction is that of what is left of *I*_NaL_ at these potential values (−40 mV *vs*. −37 mV in the control). As *G*_CaL_ increases up to its 100%, its threshold overwhelms that of *I*_NaL_ and dominates the discrete transition between voltage traces at *V*_m_ = −57 mV (right panel in **Figure 6B**). This polarized value is justified by the reactivation of this current augmented by 100%.

**Figure 7:**
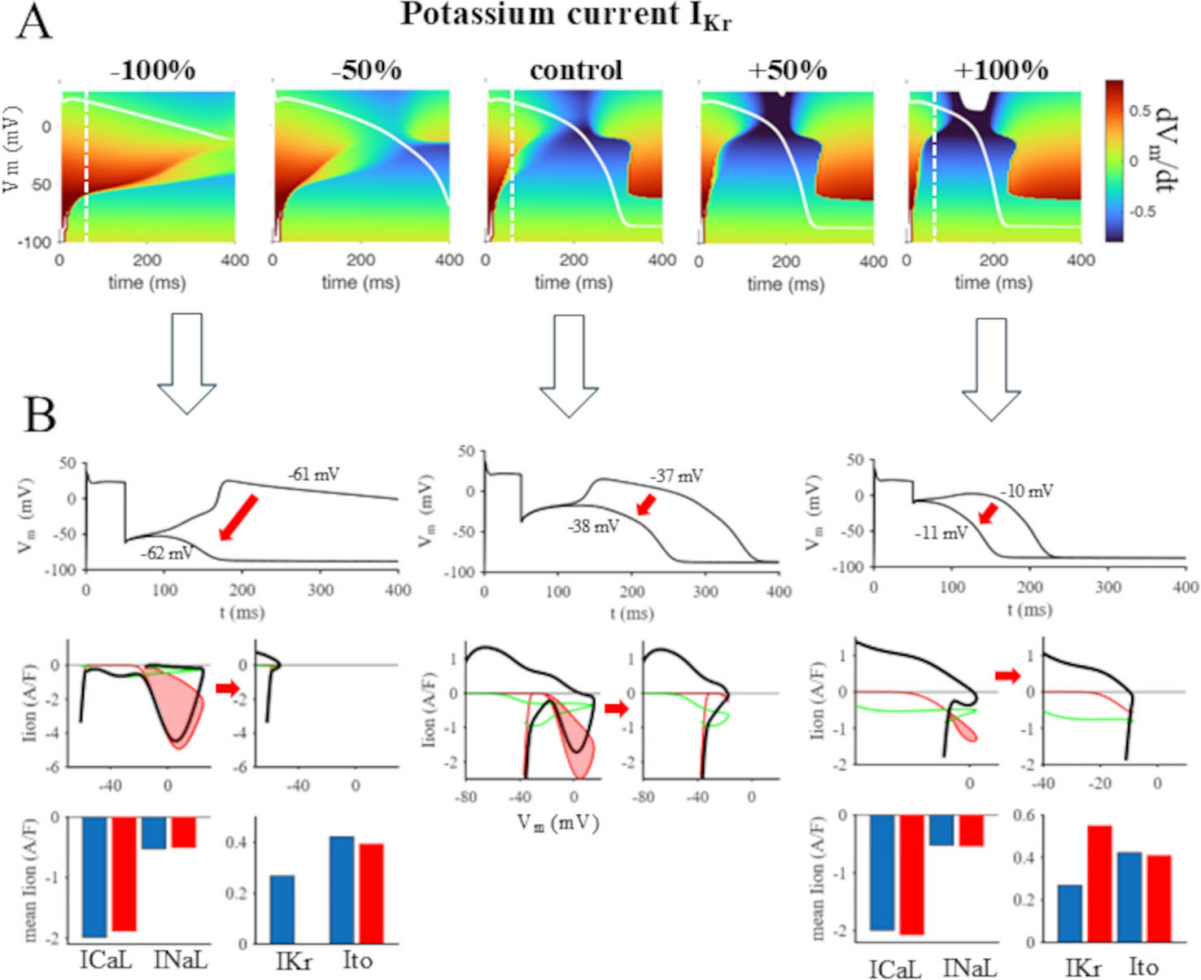
*I*_Kr_-modulation of AONR threshold. This figure is organized as the preceding one Panels in **(A)** represent how the complete abolition or the 50% decrease of IKr, or its increase by 50 or 100% modify the form of AONR thresholds. In the complete absence of *I*_Kr_, in order to switch off *I*_CaL_ re-activation, very polarized potentials have to be reached (-62 mV), where *I*_K1_ completes the final repolarization (left panel **B**). The opposite is true for 100% increase of *I*_Kr_, in which case a small polarization (-11 mV) is enough to switch off what remains at these potential values of *I*_CaL_ re-activation and leave it to *I*_Kr_ first, and *I*_K1_ then, to complete the repolarization.

Of particular interest is the role of *I*_Kr_ in modulating the threshold for AONR (**Figure 7**). In the absence of this current, the main player of self-regenerative repolarization required for completing AONR is *I*_K1_. On the other hand, in order to reach the voltage range of this current, it is necessary to turn off a fully reactivated *I*_CaL_ (see left panel in **Figure 7B**), which requires a clamp potential of −61 mV (again, compare with −37 mV in the control condition). In the case of a 100% increase in *I*_Kr_ (right in the figure), the possibility for a self-regenerative repolarization is already present at very depolarized potentials, where the threshold for AONR is still determined by *I*_CaL_, even though largely inactivated (right panel in **Figure 7B**).

**Figure 8:**
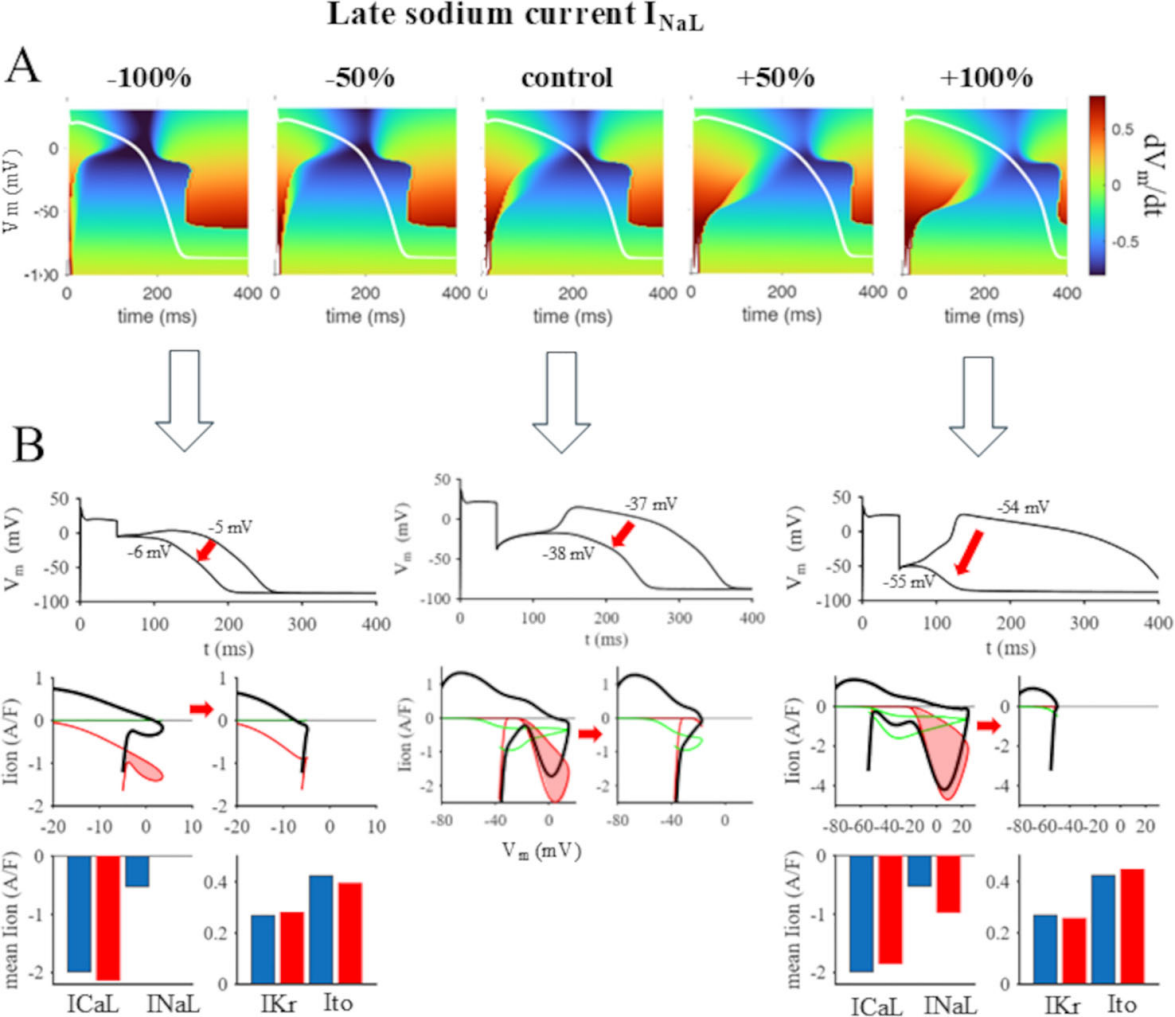
*I*_NaL_-modulation of AONR threshold. Panels in **(A)** represent how the complete abolition or the 50% decrease of INaL, or its increase by 50 or 100% modify the form of AONR thresholds. In the absence of *I*_NaL_, a very small polarization is required to reach the V_m_ value (-6 mV) required to turn off the small re-activation of *I*_CaL_ (see left panel **B**) and let *I*_Kr_ repolarize V_m_ to its resting value. In the case of a 100% increase of *I*_NaL_, a large polarization (-55 mV) is required to turn off the large re-activation of *I*_CaL_ at these potential values. Thus, despite the large increase in *I*_NaL_, at least at time T = 50 ms, the main responsible for the value of the threshold for AONR is still *I*_CaL_. At these very polarized potential values *I*_K1_ will complete the repolarization.

In the absence of *I*_NaL_ (**Figure 8**), the threshold for *I*_CaL_ is reached at a very depolarized potential where the current is partially inactivated, and what is left of its all-or-none reactivation is responsible for the threshold of AONR at these potentials (−5 mV) (**Figure 7B**). The opposite is true when *I*_NaL_ is increased by 100%, with the range where both *I*_NaL_ and *I*_CaL_ are fully reactivated being quite large and only a very polarized potential can turn *I*_CaL_ reactivation off and allow *I*_K1_ to complete the AONR (right B panel).

**Figure 9:**
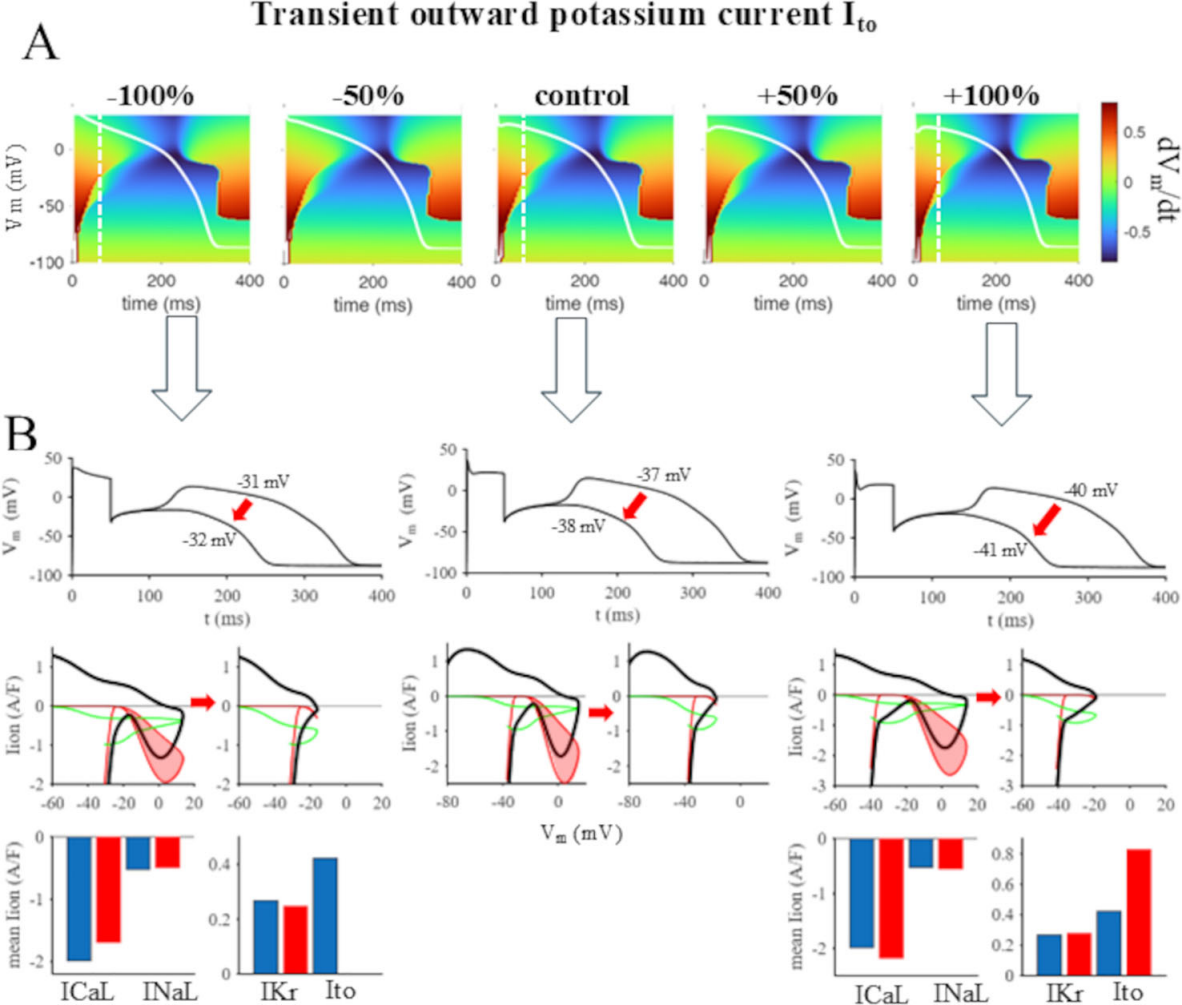
*I*_to_-modulation of AONR threshold. In the endocardial Tomek 2019 model, the role of *I*_to_ is relatively small compared with other repolarization currents and the differences introduced by large decrease or increase of this current are also relatively small. Panels in **(A)** represent how the complete abolition or the 50% decrease of Ito, or its increase by 50 or 100% modify the form of AONR thresholds. Thus, the threshold for AONR changes slightly from -100% (-31 mV) (left panel **B**), to control (-37 mV), and to 100% increase (-40 mV). In all instances, at least at time T= 50 ms, the current responsible for the threshold of AONR is *I*_CaL_ with *I*_Kr_ first and *I*_K1_ then completing the repolarization.

Among the ionic currents involved in early repolarization, *I*_to_ is the current, at least in the endocardial model, that has the smaller effect on the threshold value for AONR (**Figure 9**). As can be seen later, *I*_to_ does have a key role in AONR, particularly in the epicardial Tomek model, but in the very first milliseconds of AP and in the absence of *I*_Na_. Note, however, that the absence of *I*_to_ (**Figure 9B**) makes the very early phase of the AP more depolarized, allowing a smaller *I*_CaL_ reactivation (see lower bar plot), which in fact describes a smaller loop in the phase plot when compared with the control. For the same reason, a 100% increment of *I*_to_ leads to a slightly larger reactivation of *I*_CaL_ (right panel B). However, in this case, the presence of a larger total repolarizing current requires reaching a slightly more polarized potential (−40 mV, compared with the control) to turn off the reactivation of *I*_CaL_ and leave it to *I*_Kr_ and *I*_K1_ to complete the AONR.

**Figure 10:**
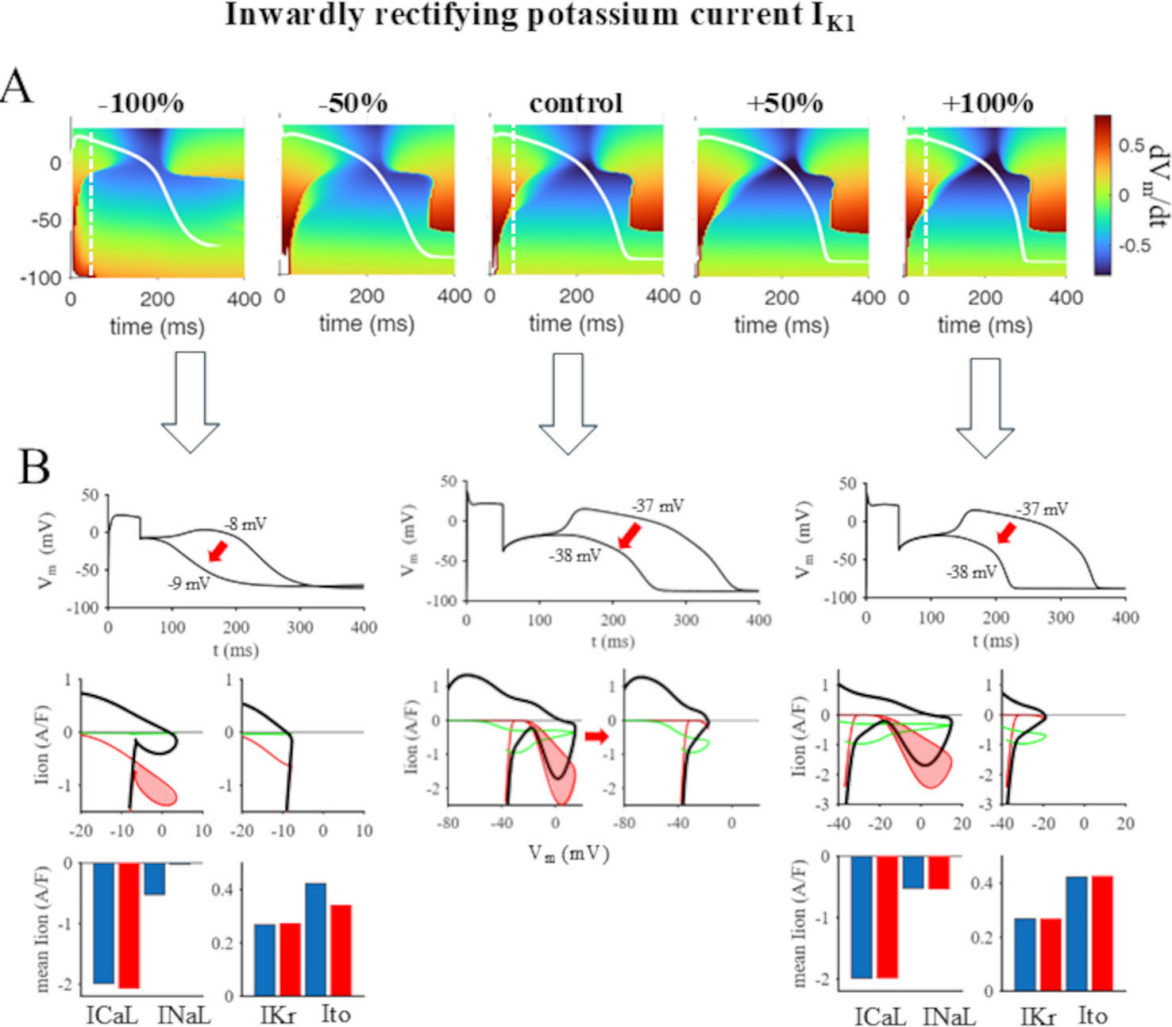
*I*_K1_-modulation of AONR threshold. Panles in **(A)** represent how the complete abolition or the 50% decrease of IK1, or its increase by 50 or 100% modify the form of AONR thresholds. The 100% decrease of *I*_K1_ causes depolarization of resting potential with consequent quasi-total inactivation of *I*_NaL_ (see bottom histogram in panel left **B**). A small polarized potential (-8 mV) is enough in this case to switch off what remains from ICaL re-activation at this potentials (left middle panel **B**). A 100% increase of *I*_K1_ does not affect, with respect to the control, the threshold for AONR (-37 mV). To note, when comparing control with +100%, the final part of post-clamp V_m_ repolarization is much steeper with +100% *I*_K1_, as expected. The initial part of post-clamp repolarization, on the contrary, is similar and mainly de to *I*_Kr_, which is the same in both instances.

Concerning *I*_K1_, the complete removal of this current shifts the threshold for AONR upwards (**Figure 10A**). The absence of *I*_K1_ depolarizes the resting membrane potential, prevents the activation of *I*_NaL_, and decreases that of *I*_to_ (lower bar plot of panel B), leaving it to the reactivation of *I*_CaL_, although partially inactivated at these *V*_m_ values, to govern the transition to AONR. See the small reactivation loop in the corresponding phase plot. Thus, a decrease in *I*_K1_ indirectly affects the threshold of AONR. Increasing *I*_K1_ does not sort any effect on the threshold of AONR (the same as in the control conditions), which is determined by the interplay between the threshold of *I*_CaL_ and *I*_Kr_, which are unaffected by this maneuver. However, *I*_K1_ does modify the shape of the voltage deflections, which are steeper in their final portion, stressing the fact that an increase in this current modulates AONR in its shape, but not in its threshold.

### Transmural gradient of AONR

**Figure 11:**
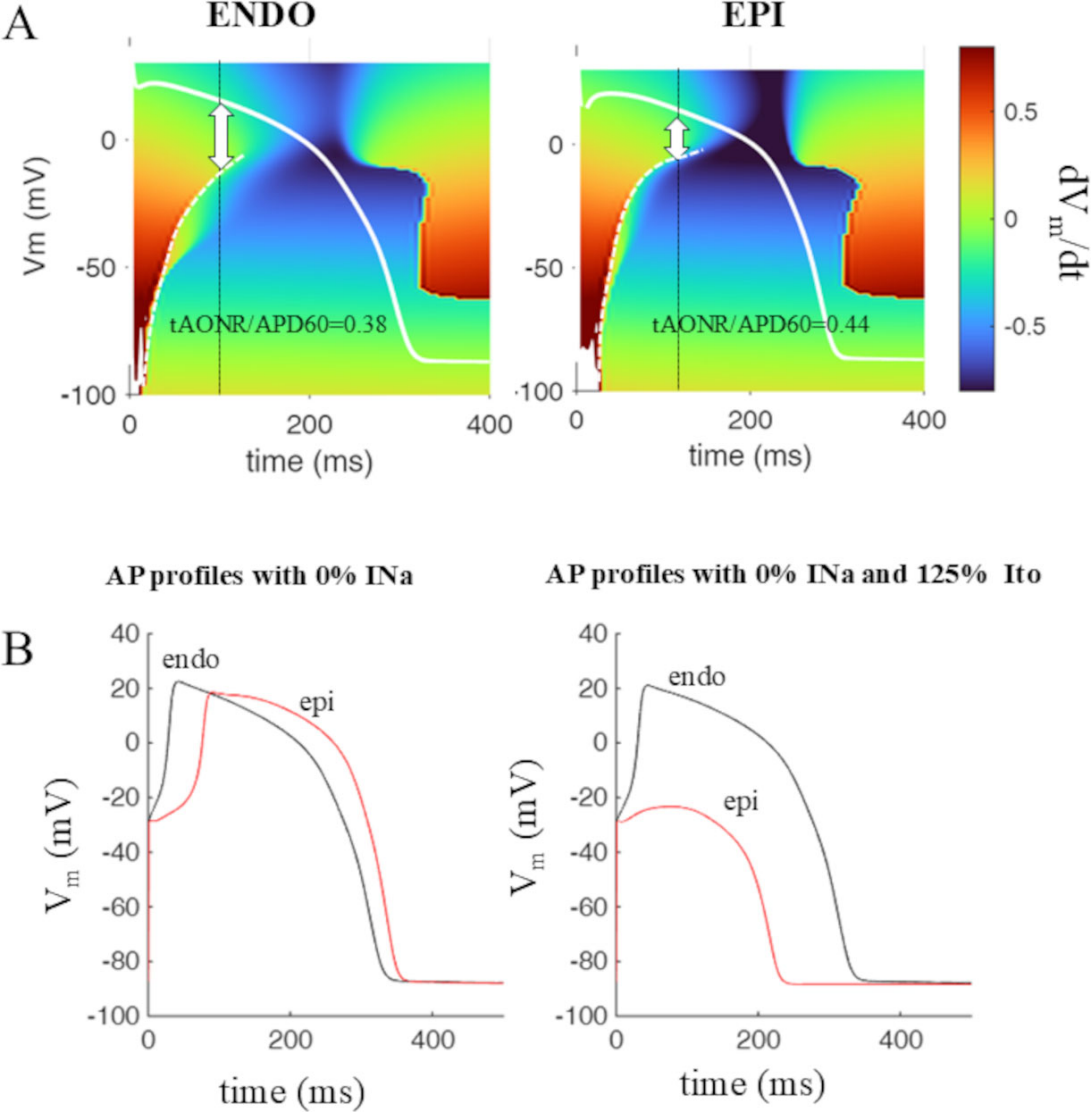
Comparison of endo and epi forms of Tomek 2019 model. **(A)** The epicardial form of the Tomek 2019 AP is characterized by a more depolarized and longer threshold for AONR with respect to the endocardial form. Note that, at T = 100 ms a much smaller polarization is needed to reach the threshold in the epicardial form, when compared with the endo (see vertical white double-arrows). **(B)** The complete removal of *I*_Na_ from both models leads to calcium-driven AP waveforms (left). When, in addition to *I*_Na_ removal, *I*_to_ was increased by 25%, the epicardial model reached spontaneously the threshold for AONR whereas the endocardial did not (right).

**Figure 11** reports the color maps corresponding to the endocardial and epicardial APs simulated at a pacing rate cycle length (CL) of 2,000 ms. The epicardial version of the Tomek 2019 model is mainly characterized by a decrease in *G*_NaL_ (−40%) and an increase in *G*_Kr_ (+30%), *G*_Ks_ (+40%), and G_K1_ (+20%), as well as with a marked increase in *G*_to_ (+400%). The combination of these changes leads to a depolarized threshold for AONR, which lasts longer (44% of APD_60_) with respect to the endocardial version (38% of APD_60_). As mentioned above, the most relevant difference in the endocardial/epicardial AONR is unmasked by the removal of *I*_Na_. As shown in the two lower panels, the removal of *I*_Na_ leads to calcium current-driven APs (**Figures 11B**, left) with a slower initial depolarization phase. When the *I*_to_ is increased by 25% in both models, the epicardial model spontaneously reaches the threshold for AONR, whereas the endocardial model does not (**Figures 11B**, right).

### Rate dependence of AONR

**Figure 12:**
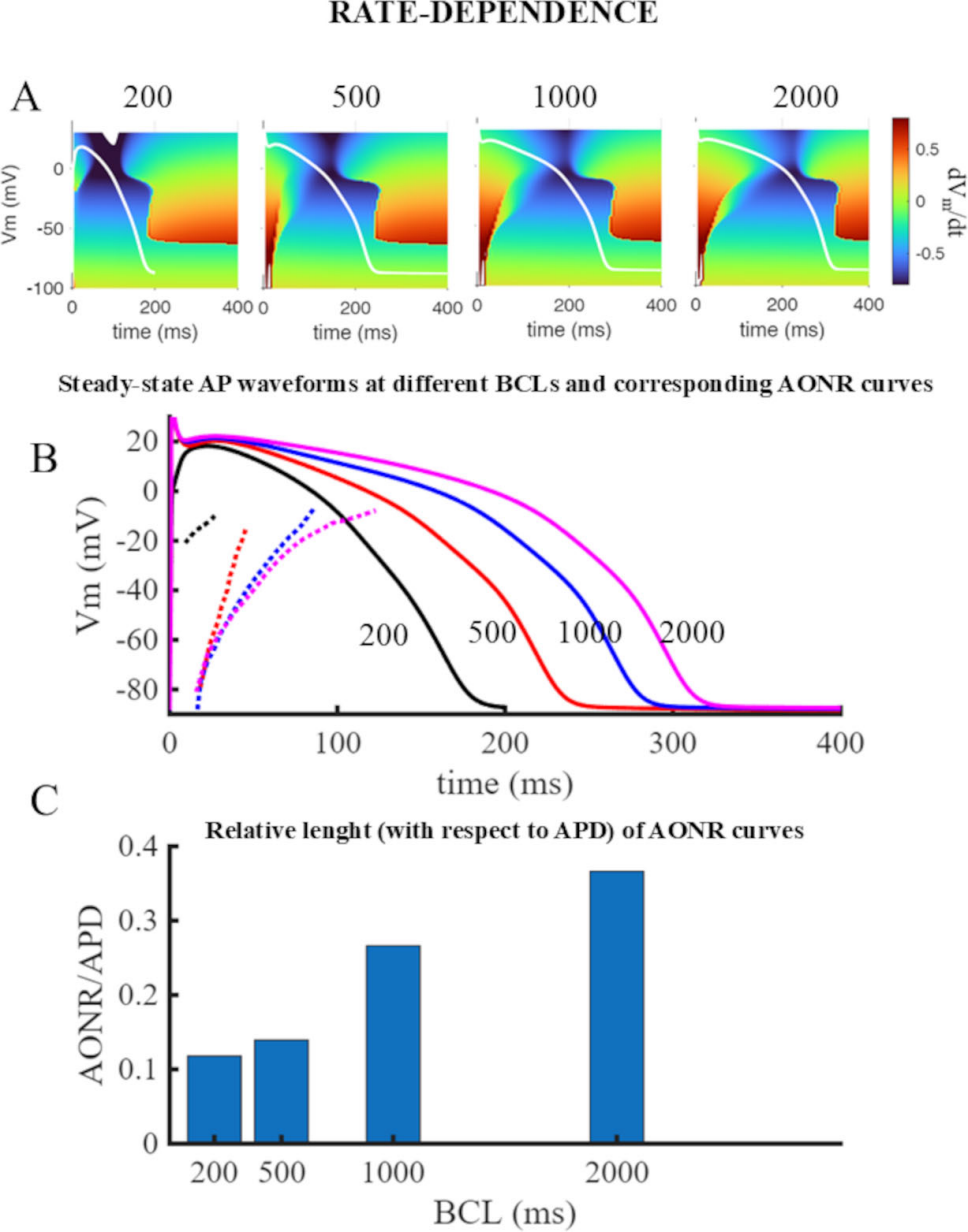
The threshold for AONR repolarization is rate dependent. The Tomek 2019 model was conditionally paced at different BCL and the three-dimensional surface measured in each case **(A)**. These show, as expected, rate-dependent AP-waveform but also quite different profiles for the threshold of AONR (see also panel **B**). When the total length of threshold for AONR was measured for each AP waveform and normalized with the corresponding APD60, it is found that the relative length of threshold increases as pacing rate decreases **(C)**.

**Figure 12A** represents four color maps obtained by pacing the Tomek 2019 model at different BCLs for 1,000 beats until the AP waveform reached steady-state configurations, the ones measured and reported in the figure. The BCL in milliseconds is reported above each panel. The profile of AONR is reported in **Figure 12B** together with the corresponding AP waveform (the same color). Apart from the BCL = 200 ms, the other AONR curves are qualitatively similar and prolonged with APD prolongation. The case of CL = 200 ms is likely due to a combination of *I*_CaL_ and *I*_NaL_ inactivation at high pacing rates and is therefore graphically similar to the condition of I_NaL_ = 0 (**Figure 3**) and *I*_CaL_ = 0 (**Figure 1**). In the lower panel, the ratio between the duration of the AONR threshold and the APD_60_ of the corresponding AP is reported. The relative length of the AONR threshold increases with increasing APD.

### Absence of AONR

**Figure 13:**
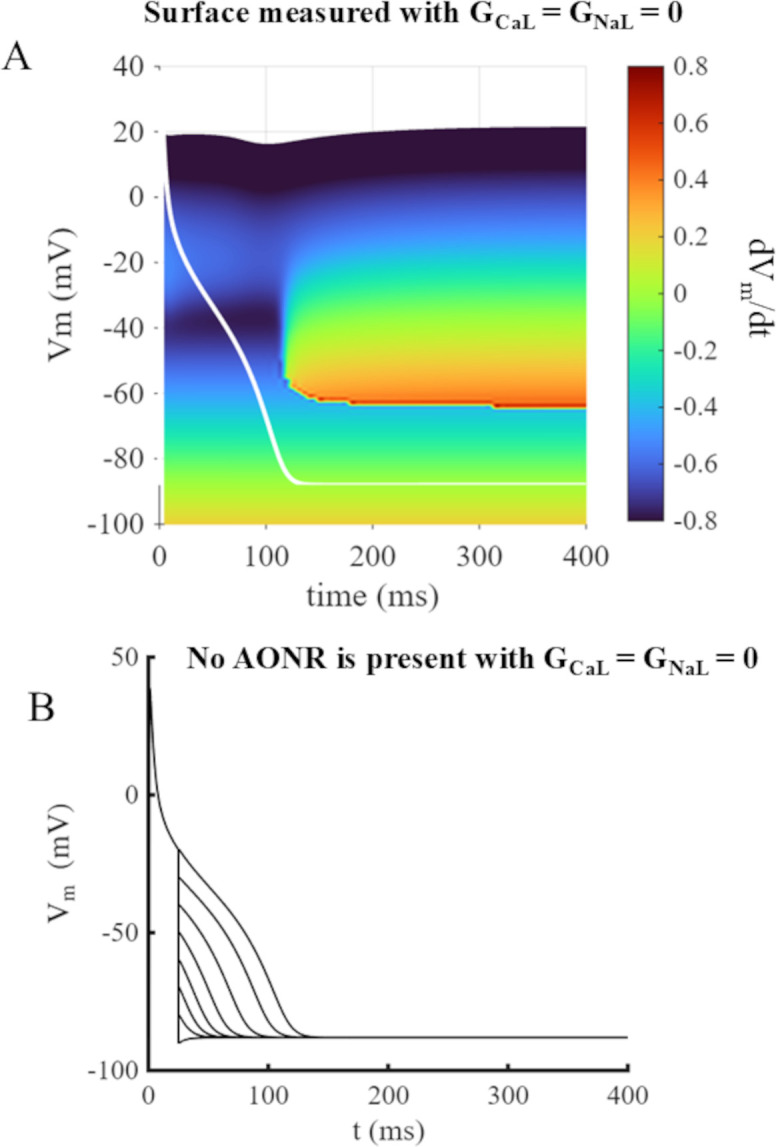
Abolition of threshold for AONR. As we have demonstrated in the previous figures, the threshold for AONR is due to the interplay between the re-activation of *I*_NaL_ and *I*_CaL_ and the self-regenerative repolarization carried out by *I*_Kr_ and *I*_K1_. We show here that the complete removal of *I*_NaL_ and *I*_CaL_ still leads to an AP, though lacking any threshold for AONR **(A)**. Accordingly, by delivering 1 ms voltage clamp steps linearly increasing (here only step 10 mV shown) in repolarizing direction at any time T during this AP (here T = 25 ms), post-clamp waveforms change smoothly in repolarizing direction without qualitative changes in their waveform **(B)**.

As it appears in the above results, the existence of a threshold for AONR is dependent on 1) the failure of a depolarizing current to reach its threshold and reactivate at a given time of repolarization and at a given potential and 2) the existence at that time and potential of one or more self-regenerative polarizing currents that bring the membrane potential to its resting value. Besides the fairly linear contribution of other depolarizing sources due to the carriers and background currents, the two main currents that can reactivate during the early phase of AP are *I*_CaL_ and *I*_NaL_, whose presence is then essential for AONR to exist. This is demonstrated by the simulation reported in **Figure 13A**, where the elimination of both currents leads to a very short AP waveform lacking AONR thresholds. Linearly increasing the voltage clamp steps (step of 10 mV) leads, in fact, to a continuous smooth modification of the post-clamp waveforms in the repolarizing direction without qualitative changes in their waveform (**Figure 13B**).

### Similar AP waveforms with a very different AONR profile

**Figure 14:**
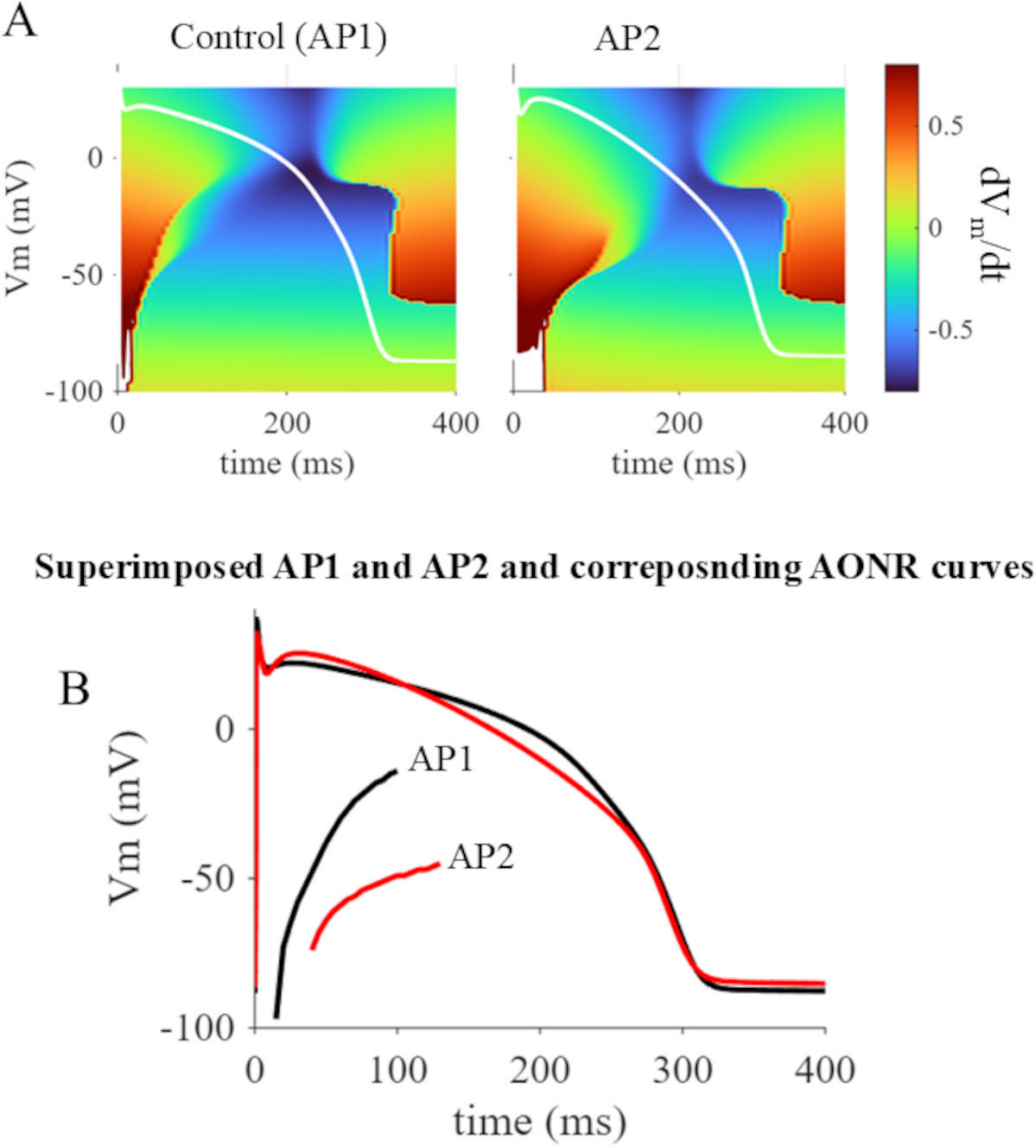
Similar AP waveforms with different thresholds for AONR. An AP waveform (AP2) has been obtained by re-modulating a number of ion currents (see text). When compared to the control Tomek 2019 AP (AP1), the two waveforms are relatively similar with identical APD60 (both waveforms are obtained by steady state pacing at BCL = 2000 ms). Despite the similarity in the AP trajectory, the two waveforms have quite different threshold for AONR [see **(A, B)**].

So far, I have examined the effect of different ion currents on the profile of the AONR threshold, although I was comparing different AP waveforms, due to the modulation of ion currents. In **Figure 14**, the case is reported of an AP obtained by modifying the *G*_max_ of a combination of ion currents within their physiological range in order to obtain an AP waveform very similar to that of the control. The two waveforms have very similar AP amplitudes, *dV*_m_/*dt*_max_, and APD. To achieve this result, the following modifications were set in the original formulation of the Tomek 2019 model: +50% of *I*_CaL_, +250% of *I*_NaL_, +50% of *I*_Kr_, −100% of *I*_Ks_, −20% of *I*_Kb_, −50% of *I*_NaK_, and +300% of *I*_Cab_. The modified AP was obtained after a conditioning pacing of 1,000 beats at CL = 2,000 ms. The corresponding color map (AP2) is reported in **Figure 14A** together with the control (AP1) obtained at the same CL. The two similar AP waveforms are reported in **Figure 14B**, together with the corresponding AONR profiles derived from the color maps above. From what I have shown above, the AONR threshold represents the value of the membrane potential that has to be reached to start a self-regenerative repolarization of the membrane after the threshold for *I*_CaL_ or that of *I*_NaL_ is exceeded. Such value is more polarized in the case of AP2 with respect to the control AP1.

## Discussion

Despite the conspicuous literature covering AONR and its propagation, a systematic description of the profile of AONR in control conditions and under ion current modulation is lacking, as there is a lack of studies on how the pacing rate modifies AONR and how AONR can affect AP propagation between cardiac cells. Knowledge of these properties points to AONR as a key biomarker in the process of developing new human ventricular AP models or of tuning those that already exist.

**Figures 1**–**3** show that the AONR profile during an AP can be measured by means of a relatively simple stimulation protocol. Whereas the color map reported in **Figure 3** requires a combination of current and voltage clamp and some data processing, which are suited here to explain its properties, the AONR profile can be easily derived by brief current injection during AP repolarization (**Figure 1**). On the other hand, color maps such as that derived in **Figure 3** allow precise measurements on the characteristics of the phenomenon. They allow, for example, appreciating the dramatic difference between the AONR profile measured in different models of the human ventricular AP (**Figure 4**). Despite the ease in its measurement, AONR is highly underestimated as a biomarker for characterizing different AP models. Many studies compare different human ventricular AP models for their ability to reproduce real physiopathological conditions (Bueno-Orovio et al., 2008; Cummins et al., 2014; Dong et al., 2025; Elshrif and Cherry, 2014; Roshan and Pandit, 2026; Ten Tusscher et al., 2006; Tomek et al., 2019; Trayanova et al., 2024; Tomek et al., 2025), although none of them has taken AONR into consideration. The waveform of the AP and the rate dependence and restitution of APD, together with the usual AP biomarkers including *dV*_m_/*dt*_max_, AP amplitude, AP notch, *V*_rest_, the BCL-inducing alternans, and many others, are frequently compared in the works referenced above, but not the AONR threshold, and this despite the fact that such parameter expresses concisely the fine-tuning between the depolarizing and polarizing currents during the course of the early AP phase, which is fundamental for the AP dynamics and does not result trivially from the other AP parameters. To this, it should be added that, in many of these comparative works, one aspect that emerges is the sometimes dramatic difference between the relative role of the repolarizing ion currents, particularly *I*_Kr_ and *I*_Ks_ (Bueno-Orovio et al., 2008; Dong et al., 2025), but also *I*_CaL_ (Roshan and Pandit, 2026), which, as I have shown, are key players in determining the shape of the AONR threshold.

Although partially described elsewhere (Trenor et al., 2017; Himeno et al., 2023), I try here to resume the mechanism of the emergence of a threshold for AONR based on the results presented above and mainly focusing on the most significant ionic players. Right after the onset of the AP, repolarizing currents start to activate, particularly *I*_to_ and *I*_Kr_, and, only later, *I*_K1_. Concurrently, a number of depolarizing currents, almost fully activated at the onset of AP, start to slowly inactivate along the course of the AP. When the membrane potential is suddenly brought to a more polarized value, either in the current or the voltage clamp, this tends to deactivate the depolarizing currents including *I*_CaL_ and *I*_NaL_. These, when not completely time-dependently inactivated, tend to reactivate along their negative-sloping, self-regenerative, *IV* curve, as soon as the current injection or the voltage clamp is released. I recall that a negative *IV* curve generates, as *V* changes, a variation in *I* that further increases the *V* change, thus leading to positive feedback that reinforces *V* variation. The amount of reactivated depolarizing current also depends on the electromotive force for that current at the membrane potential reached during current injection/voltage clamp. The threshold for AONR is determined by the all-or-none failure of these depolarizing currents to reactivate, which leaves it to the self-regenerative potassium currents (negative-sloping *IV* curve of *I*_Kr_ and *I*_Ks_) to complete the repolarization. The contribution to AONR of the self-regenerative depolarizing currents decreases with time as they inactivate during the course of the AP. With their inactivation, the threshold for AONR tends to disappear, leading the current injection/voltage clamp-induced polarizations to simply repolarize the membrane potential with a time course linearly dependent on their amplitude. I analyze only the contribution to AONR of certain ion currents, intentionally avoiding, for the sake of clarity, those ion currents whose contribution is smaller and, most frequently, linear. Thus, I find the depolarized thresholds for AONR in **Figure 7** (right panel; +100% *I*_Kr_), in **Figure 8** (left panel; −100% *I*_NaL_), or in **Figure 10** (left panel; −100% *I*_K1_), where the reactivation of *I*_CaL_ is minimal, although enough to generate the all-or-none response. On the contrary, in **Figure 6** (right panel; +100% *I*_CaL_) and **Figure 7** (left panel; −100% *I*_Kr_), I find very polarized thresholds with full reactivation of *I*_CaL_. Thus, the shape of AONR depends on the fine balance of the ion currents, particularly *I*_CaL_, *I*_NaL_, and *I*_Kr_, involved in human AP plateau and therefore is rate-dependent (**Figure 11**). The existence of a threshold for AONR requires depolarizing currents that have a threshold for activation; accordingly, the absence of such currents leads to an AP without a threshold for AONR (**Figure 13**).

The case reported in **Figure 11** summarizes the relevance of the threshold for AONR. **Figure 11A** shows that more energy (white double arrows represent either current injections or voltage clamps) is needed, for example at *T* = 100 ms, to reach the threshold for AONR in the endocardial than in the epicardial Tomek 2019 AP type. In **Figure 11B** (left), *I*_Na_ is completely abolished, mimicking the action of a class I anti-arrhythmic agent, which leads to the calcium current-driven APs reported in the figure. When, in addition, *I*_to_ is increased in both models by 25%, the epicardial cell reached spontaneously, right after pacing, the threshold for AONR, while the endocardial cell repolarized along its unperturbed waveform (**Figure 11B**). When a circumstance such as that in **Figure 11B** would happen *in vivo*, a gradient of *V*_m_ would be established in the tissue between the endocardium and the epicardium, which has been proposed to cause reentry potentially leading to tachycardia or arrhythmias (Antzelevitch et al., 1999). The polarization levels considered here can, for example, result from the use of pinacidil, which has been proposed as a pharmacological approach in the treatment of arrhythmias (Tseng and Hoffman, 1990). Among other effects, pinacidil in fact is known to augment the ATP-regulated potassium current, *I*_K-ATP_, in cardiac tissue (Carlsson et al., 1992), causing early polarization of the AP waveform. Similarly, global ischemia can cause AONR at the epicardium, but not at the endocardium, causing transmural gradient of repolarization, ST segment elevation, and extrasystolic activity secondary to phase 2 reentry (Di Diego and Antzelevitch, 2003).

One of the reasons for AONR often being ignored is that it involves a range of *V*_m_ values that are normally not reached during the physiological course of an AP. However, there are indeed circumstances in which this time/*V*_m_ region becomes significant, e.g., during the discontinuous conduction of the AP. During discontinuous AP conduction, in fact, the membrane potential of the sink cell is brought into a time/voltage range where the existence and the form of a threshold for AONR are highly relevant. Heterogeneous spatial dispersion of electrophysiological properties, even in the absence of a difference in the AP waveform, can lead therefore to discontinuous conduction, which, at the whole organ level, is known to cause fragmented electrograms in patients with sustained ventricular tachycardia in the presence of coronary artery disease (Josephson et al., 1997). In addition to the effect on discontinuous AP conduction, the data in **Figure 14** also remark the fact that the existence and the form of AONR cannot be simply predicted from the AP waveform.

## Conclusions

The form of the threshold for AONR can be easily derived in current clamp or voltage clamp mode and does not depend trivially on the AP waveform. The form for AONR depends on the fine balance between the ion currents active in the early AP repolarization phase and results to be different in different human ventricular AP models. The AONR is chiefly determined by the reactivation of inactivating depolarizing currents such as *I*_CaL_ and *I*_NaL_ and by the final contribution of self-regenerative repolarization currents such as *I*_Kr_ and *I*_K1_. The AONR form depends on the pacing rate. The difference in the form and strength of AONR in epicardial and endocardial myocytes can induce transmural gradients of repolarization that can result in reentry and arrhythmias. The form and the polarization level of the threshold for AONR do not depend on the form of the AP waveform and can play a role in the discontinuous conduction of AP. All these findings suggest that the threshold for AONR is highly suited as a biomarker to be taken into consideration when fine-tuning the ion currents in the process of developing and improving human ventricular AP models.

Matlab Code for calculating color maps can be found at Zenodo public repository https://doi.org/10.5281/zenodo.19666107.

## Data Availability

The raw data supporting the conclusions of this article will be made available by the authors, without undue reservation.
